# Effects of physical training on functional, clinical, morphological, behavioural and psychosocial outcomes in post-COVID-19 infection: COVID-19 and REhabilitation study (CORE-study)—a study protocol for a randomised controlled clinical trial

**DOI:** 10.1186/s13063-022-07055-5

**Published:** 2023-01-19

**Authors:** Rodrigo Sudatti Delevatti, Angelica Danielevicz, Maria Eduarda Sirydakis, Paulo Urubatan Gama de Melo, Cíntia de la Rocha Freitas, Cassiano Ricardo Rech, Luiz Guilherme Antonacci Guglielmo, Guilherme Fleury Fina Speretta, Fernanda Hansen, Fernanda Rodrigues Fonseca, Ana Carolina Starke, Ricardo Dantas de Lucas, José Tavares de Melo Junior, Rosemeri Maurici, Aline Mendes Gerage

**Affiliations:** 1 Department of Physical Education, Sports Center, Federal University of Santa Catarina, University Campus, Trindade, Florianópolis, Santa Catarina 88040-900 Brazil; 2 Department of Nutrition, Health Sciences Center, Federal University of Santa Catarina, University Campus, Trindade, Florianópolis, Santa Catarina 88040-900 Brazil; 3 Health Sciences Center/NUPAIVA, Federal University of Santa Catarina, University Campus, Trindade, Florianópolis, Santa Catarina 88040-900 Brazil

**Keywords:** COVID-19, Rehabilitation, Physical training, Multicomponent exercise

## Abstract

**Background:**

The COVID-19 pandemic remains ongoing, with a significant number of survivors who have experienced moderate to severe clinical conditions and who have suffered losses of great magnitude, especially in functional capacity, triggering limitations to daily autonomy and quality of life. Among the possibilities of intervention for disease rehabilitation, physical exercise training stands out, which can benefit several health outcomes and favours the adoption of healthier behaviours. Therefore, the aim of the study will be to analyse the effects of physical training on the functional, clinical, morphological, behavioural and psychosocial status in adults and the elderly following COVID-19 infection.

**Methods:**

A randomised controlled clinical trial is to be conducted in parallel, with the experimental group undergoing an intervention involving a multicomponent physical rehabilitation programme, carried out at the Sports Center in partnership with the Academic Hospital of the Federal University of Santa Catarina, in Florianópolis, Brazil. Participants will be adults and the elderly, of both sexes, in a post-COVID-19-infection state, who were hospitalised during the infection. The intervention will have a total duration of 24 weeks and will include a multicomponent physical training programme, which will have gradual progression in frequency, duration and intensity over time. Regarding the outcomes, before, at the 12th and after 24 weeks of intervention, functional (primary outcome = functional index of aerobic capacity), clinical, morphological, behavioural and psychosocial outcomes will be assessed.

**Discussion:**

This study will contribute to a greater understanding of the safety, adherence and benefits of physical training in the rehabilitation of post-COVID-19 patients. The results of this study will be disseminated through presentations at congresses, workshops, peer-reviewed publications and local and international conferences, especially with a view to proposing a post-COVID-19 rehabilitation care protocol.

**Trial registration:**

ReBEC, RBR-10y6jhrs. Registered on 22 February 2022. 2015.

**Supplementary Information:**

The online version contains supplementary material available at 10.1186/s13063-022-07055-5.

## Introduction

Among the various viruses that cause upper airway infections, influenza A, rhinovirus and coronavirus stand out, being present in more than 70% of cases of respiratory infections, which makes it necessary for greater knowledge and effective therapy against these diseases. Respiratory infections of viral etiology are considered a public health problem, causing a high and constant increase in mortality rates worldwide [1]. These diseases are highly communicable and pose a risk to the population, even though they are linked to the environment and are more preventable than diseases in other organ systems [1]. Outbreaks, such as those of severe acute respiratory diseases (SARD) already existing in human history, show that there are environmental and individual factors that people can adopt to protect themselves and others [2].

More than one million people became ill with the H1N1 flu between April and June 2009 in the USA, with cases with complications, with the development of severe pneumonitis and SARD and with consequent multiple organ failure. Three months after hospital discharge for SARD, up to 80% of patients demonstrated reduced diffusion capacity as well as reduced quality of life [3].

In this condition, post-H1N1 with complications [4] found improvements in pulmonary function, functional capacity and quality of life after 2 months of a multicomponent training (resistance, respiratory and aerobic) in patients previously hospitalised. However, in January 2020, something completely new emerged, which the World Health Organization (WHO) declared a public health emergency: the disease caused by the novel coronavirus (COVID-19). In March of that year, it was characterised as a pandemic, with significant global impacts, especially on health and the economy [5, 6]. The disease known as COVID-19 is characterised by a viral infection caused by the type 2 coronavirus (SARS-CoV-2), providing a varied and complex clinical scenario, with likely impairment in several organ systems, which can present from mild to moderate signs and symptoms up to a picture of severe acute respiratory syndrome [5, 7].

The possible clinical manifestations in patients with COVID-19 are wide and include fever, cough, breath shortness, dyspnea, appetite loss, nausea, vomiting, diarrhoea, muscle pain, general fatigue, headache, smell or taste dysfunction and mental confusion. Furthermore, the disease can progress to more serious complications, including severe acute respiratory syndrome (SARS), acute kidney injury, anaemia, heart failure and secondary infection [8].

In Brazil, as occurred in different regions of the world, the health organisations, especially the public system, were largely affected by the COVID-19 pandemic. The fight against this pandemic condition basically takes place in three types of action: (a) the prevention of contagion throughout the promotion of preventive actions, such as social distancing, use of masks, adequate hand hygiene and immunisation strategies in the national territory [5]; (b) treatment of patients during the viral cycle through therapies according to the level of impairment, aiming to reduce the chance of sequelae and, above all, mortality [9]; (c) rehabilitation of patients after the viral cycle, aiming at the recovery of the different organic systems affected to reintegrate people with autonomy and quality of life into society [8].

The current scenario demonstrates that the number of survivors is highly expressive, especially those who experienced moderate to severe clinical conditions in the acute phase of the disease, who suffered losses of great magnitude, especially in functional capacity, limiting autonomy and quality of life. In this context, the literature has presented a clinical picture in which people manifest continuous symptoms after their initial COVID-19 infection, called “long COVID” or “post-COVID syndrome”. This syndrome is a multisystem condition with a number of debilitating symptoms, including fatigue, dyspnea, cough, chest pain, headache, muscle pain and gastrointestinal problems, among others. In addition, people with “long COVID” can experience psychological and cognitive problems, such as depression, anxiety and post-traumatic stress disorder. These symptoms can result in significant impacts on the subject’s ability to perform their daily and even work tasks [10–12].

Given this varied profile of persistent symptoms chronically impacting people’s lives, interventions are necessary that at least minimise the negative impact of the disease, especially on functional capacity. For this, the need for research into the role of physical exercise in the rehabilitation of patients, given the great number of benefits of exercise in the rehabilitation of other frail populations, is understandable. In post-COVID rehabilitation, a review by Sun et al. [8] demonstrated benefits such as improved patient awareness, reduced mechanical ventilation time, improved respiratory function and reduced risk of complications, mortality rates and risks of readmission. Furthermore, according to the authors, in the case of patients who have COVID-19 in whom it is manifesting in a severe and critically ill manner, rehabilitation should only begin when the patient’s condition is stable. Among the exercises proposed for rehabilitation in the studies that made up the review, there are respiratory exercises, postural maintenance and control, active and passive joint mobility, standing and standing independently. Still, some studies have reported benefits of conventional [13] and unconventional exercise models [14] in post-COVID patients.

Although the number of scientific investigations on the topic of post-COVID rehabilitation has been growing, to date, few studies have precisely investigated the effects of physical rehabilitation in patients who have had moderate to severe COVID-19. Furthermore, the recent literature on physical training in post-COVID-19 rehabilitation is still lacking in terms of multicomponent protocols (a combination of two or more components, such as aerobic, resistance, balance and flexibility exercises in the same training session) in a progressive approach to the variables (volume and intensity) of training. A multicomponent approach is important due to multisystemic losses found in this population. In this direction, it is important to intervene in the main physical capacities impacted by the disease and associated with functional losses. Multicomponent training has been shown to be the most beneficial type of exercise to improve the functional capacity of frail older people [15–17]. Also, as the post-COVID-19 condition is still not completely understood, presenting a certain heterogeneity among patients to provide training for multiple physical capacities is more prudent than a more specific approach regarding the type of training.

Thus, the objective of this study is to describe the methodology used in a randomised clinical trial that seeks to analyse the effects of a rehabilitation programme on clinical, functional, morphological, behavioural and psychosocial outcomes in patients who were infected with SARS-Cov-2.

## Methods

### Design

This is a randomised controlled trial (RCT), of superiority and unicentric, conducted in parallel with blinding of outcome assessors. The study will have two arms: a multicomponent physical training arm, with progression in volume and intensity, and a control arm, without structured exercise. The study is registered in the Brazilian Registry of Clinical Trials (code RBR-10y6jhrs).

### Setting

All procedures of this study will be performed at the Federal University of Santa Catarina (UFSC), located in Brazil. Recruitment and eligibility, as well as the assessment of several outcomes, will take place at the Asthma and Airway Inflammation Research Centre (NUPAIVA) at the University Hospital at UFSC facilities. The rehabilitation programme (intervention) and the evaluation of other outcomes will be carried in the facilities at the sports centre UFSC. Among these facilities, there are three other places for the exercise intervention, namely the rehabilitation centre, the strength training room and the athletics track and field, all located at UFSC.

### Participants

The whole sample (including men and women > 18 years old) who were hospitalised for the treatment of COVID-19 and who experienced moderate or critical conditions in the acute phase of infection, will participate in this study.

Patients who have been discharged from hospital for at least 6 weeks and who meet the following criteria will be included: 72 h afebrile without the use of antipyretics and stabilisation of respiratory symptoms; absence of mechanical ventilation and tracheostomy devices; absence of hypersecretion with ineffective cough; absence of severe dyspnea, difficult to stabilise, at rest and in activities of daily living; respiratory rate < 18 breaths per minute; peripheral O_2_ saturation (SpO_2_) > 90%; normal resting electrocardiogram (12-lead); control of underlying diseases (under medical supervision); absence of open lesions; ability to sit and stand without assistance; ability to maintain balance in the standing position; and a stable level of consciousness and absence of mental confusion. Besides that, at the beginning of the research, patients will not be able to engage in exercise programmes with a focus on aerobic, resistance or balance components, at a frequency of twice a week or more.

The selection of participants will occur on a voluntary and non-probabilistic basis. Participants will be recruited from a list of patients who had treatment at the University Hospital and will be provided by the NUPAIVA team of physicians and physiotherapists. From this list, the research team of kinesiology professionals will invite the potential participants via phone call or messaging app.

If sample *n* is not reached, media dissemination will be carried out, aiming to reach patients who were hospitalised in other hospitals. Also, if the sample *n* is not reached only with hospitalised patients, the recruitment will be open to patients who, after infection by COVID-19, present chronic fatigue, moderate to severe, as evaluated by the Chalder scale [18].

**Fig. 1:**
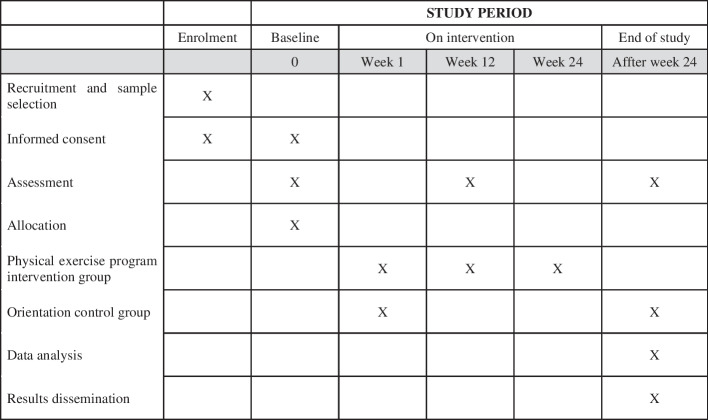
Schedule of enrolment, interventions and assessments

All eligible patients interested in participating in the current study will undergo a medical screening, consisting of clinical evaluation and ECG analysis. Once the patients are allowed to exercise, the research team will schedule a meeting to explain the study procedures and for the signing of the informed consent form. For those who consent to participate, a complementary anamnesis will be applied to the medical screening, with a primary focus on lifestyle, and later the battery of tests for baseline assessment will be scheduled (Fig. 1).

### Sample size calculation

The sample size calculation was based in an RCT [19] with a population and design similar to the present study. The calculation was performed for the distance covered in the 6-min walk test, the primary exercise outcome of the present study. This aforementioned study showed within-group and between-group changes (i.e. improvement of 50 m on the distance covered) after intervention in favour of the experimental group, which would be expected in the present study. The calculation was performed using the G*POWER 3.1 programme, adopting a significance level of 0.05, a power of 80% and a correlation coefficient of 0.5. The resulting *n* is 22 participants in each group. Considering a likely sample loss of around 30%, the recruitment will aim to reach an *n* sample of 30 participants for each group, totalling around 60 participants. The expected flow of participants into the trial is six to eight participants (blocks) every 15–25 days.

### Intervention

The intervention will last 24 weeks and will consist of two weekly training sessions in the first 11 weeks, reassessments in the 12th week and three weekly sessions from the 13th to 24th weeks. The planned training will have two major stages, the first consisting of two mesocycles, with progression primarily in volume, and the second consisting of three mesocycles, with progression primarily in the intensity (Table 1).

**Table 1:** Structuring of training sessions throughout the Rehabilitation Program (CORE-Study)

**Weeks**	**Structure (Component-type, volume, intensity, progression)**
1	Familiarisation with the training model (focus on learning movements and perceived effort)
2 a 6	**Balance**—3 exercises performed in three sets of 10–30 s, with evolution in time (10 to 30 s) and complexity (stages). Unipedal balance—standing on one foot, balance transitioning from dorsiflexion to plantar flexion, and getting up from a chair and walking in a straight line. The stages are (1) Support from both hands (chair/instructor/wall); (2) One-handed assistance (chair/instructor/wall); (3) No assistance; (4) With eyes closed. **Aerobic (treadmill walking)**—total: 25 min accumulating 15 min of light intensity (RPE: 12/13) and 9–10 min of passive recovery). Duration of blocks in two levels—5 repetitions of 3-min walk with 2-min passive intervals, or 3 repetitions of 5-min walk with 3-min passive intervals. **Resistance (body weight and rubber band)**—Stretch bench press, Sit and Stand, Neutral Rubber Row, Step Up and Down Step and Plantar Flexion (2 sets of 10 to 15 reps at RPE 12/13, interval of 1 min).
7 a 11	**Balance**—3 exercises performed in three sets of 10–30 s, with evolution of time (10 to 30 s) and complexity (stages). Unipedal balance—standing on one foot, balance transitioning from dorsiflexion to plantar flexion, and getting up from a chair and walking in a straight line. The stages are (1) Support from both hands (chair/instructor/wall); (2) One-handed assistance (chair/instructor/wall); (3) No assistance; (4) With eyes closed. **Aerobic (Treadmill walking)**—total: 25 min (20 min at light intensity (RPE: 12/13) interspersed with 5 min of passive recovery). Duration of blocks in two levels—5 blocks of 4 min of walking with passive 2-min intervals, or 4 blocks of 5-min walking with 2-min passive intervals. **Resistance (body weight and rubber band)**—Stretch bench press, Sit and Lift, Neutral Rubber Row, Step Up and Down Step and Plantar Flexion (3 sets of 10 to 15 reps at RPE 12/13, interval 1 min)—same setting from above.
12	Reassessments of the outcomes from Functional Capacity, Anthropometry and Body Composition, Cardiovascular Parameters and Psychosocial Parameters.
13 a 16	Aerobic (Walking—treadmill or athletic track*)—Continuous training—25 min at moderate intensity (RPE: 12/13). **Resistance (machines and free weights)**—Horizontal Leg Press, Sitting Row, Flexion Chair Knee Flexion, Vertical Peck Deck, Plantar Flexion with Back Barbell (3 sets of 12 to 15 RM, 1 min interval)
17 a 20	**Aerobic** (Walking - treadmill or athletic track*)—Interval training—5 repetitions of 5 min (1 min at RPE 15 with 4 min at RPE 12/13). **Resistance (machines and free weights)—**Horizontal Leg Press, Seated Row, Flexion Chair Knee Flexion, Vertical Peck Deck, Plantar Flexion with Barbell on the Back (3 sets of 10 to 12 RM, 1 min interval).
21 a 24	Aerobic (Walking—treadmill or athletic track*)—Interval training—6 repetitions of 4 min (1 min at RPE 15 with 3 min at RPE 12/13). **Resistance (machines and free weights)—**Horizontal Leg Press, Seated Row, Flexion Chair Knee Flexion, Vertical Peck Deck, Plantar Flexion with Back Barbell (3 sets of 8 to 10 RM, 1 min interval)

*RPE* rate of perceived effort, *reps* repetitions, *MR* maximum repetitions

*Dependent on climatic factors and the clinical condition of the patients

In stage 1, the sessions will be held at the rehabilitation centre, with a session duration of approximately 60 min, consisting of a joint warm-up (5 min) followed by a balance section (10 min), aerobic training section (25 min), resistance training (15 min) and final stretching (5 min). The order of the main parts (aerobic and resistance) will be alternated over the weeks to reduce their monotony and not prioritise one of the components, in view of the concurrent interference effect that these types of training can exert on each other.

In stage 2, some aerobic training sessions can be performed on the athletics track, depending on the weather conditions, desire and clinical condition of the participants, while the resistance training part will take place in the university gym room.

**Fig. 2:**
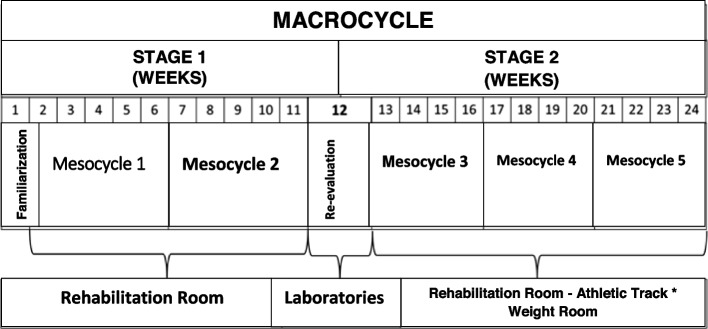
Temporal structure of the intervention (Rehabilitation Program - CORE-Study)

Three instructors with prior experience with exercise prescription will supervise all the sessions. The programme will also have two physical education students, who will assist in the general procedures of the intervention, from the reception of participants and maintenance of adequate sanitary conditions to specific assistance for the sessions that teachers may need. The performance of a concomitant structured exercise training intervention is prohibited during the trial, except physiotherapy intervention of short duration, which is permitted Fig. 2.

### Adherence

The number of sessions completed and the number of sample losses will be recorded and considered in the analysis and discussion of the results. As strategies to optimise adherence, the present trial presents a wide range of schedules available for carrying out the intervention. In addition, affectivity to exercise will be monitored at some points during the intervention. WhatsApp groups will be created with patients and care/intervention providers to facilitate communication, and telephone contact with management (first moment) or coordination (second moment) will occur when two or more consecutive absences from the intervention occur. In these calls, the focus will be on getting to know the patient’ clinical situation and asking if there is anything that the research team can help with in order to maintain the follow-up in the study.

### Control procedure

Patients allocated to the control group will receive recommendations about physical activity and sedentary behaviour. For this, two chapters from the Brazilian Guide to Physical Activity [20] will be provided and explained to patients during a personal meeting. One chapter is entitled “Understanding Physical Activity”, and the other procedural chapter is entitled “Physical Activity for Adults” or “Physical Activity for the Elderly”, depending on the age group of the participants.

### Outcomes

The evaluation of all outcomes will be blinded and performed by experienced researchers. In addition to being evaluated at baseline, the outcomes of functional capacity, anthropometrical and body composition, cardiovascular and psychosocial parameters will be reassessed at the 12th and after 24 weeks of the rehabilitation programme. Other parameters derived from the neuromuscular, respiratory, cardiorespiratory and behavioural assessments will only be carried out at baseline and at the end of the intervention period (after 24 weeks), due to logistical reasons.

### Primary outcomes

The primary outcome was the functional indicator of cardiorespiratory fitness after 24 weeks assessed by the 6-min walking test (6MWT), following the protocol of the American Thoracic Society [21] and recorded as 6MWD in meters.

### Secondary outcomes

As secondary outcomes, indicators of functional capacity, neuromuscular parameters, anthropometrical and body composition, respiratory, cardiovascular, cardiorespiratory, behavioural and psychosocial parameters will be evaluated.

### Characterisation variables

Patients included in the study will respond to an anamnesis containing sociodemographic information (sex, age), clinical history related to the period of COVID-19 infection (need and duration of ICU hospitalisation, complications acquired in the hospital environment, need for oxygen and ventilation) and general health (comorbidities, medication use, history of injuries and/or surgeries). In addition, information regarding the patient’ exercise background will be collected.

### Safety and training monitoring

As monitoring and safety variables, information on external and internal training load, SpO_2_, heart rate, blood pressure, capillary blood glucose and affective response to exercise will be collected. The internal load of each training session will be quantified by the session RPE method (RPE session), using the adapted Borg CR1 scale [22] in order to estimate the intensity after each exercise session. For such measurement, it will be asked, individually, how was the perceived intensity of the training session. In addition to RPE, heart rate (HR) will be recorded during and after the aerobic section of training, being another parameter of internal load and important for hemodynamic safety.

The quantification of the external load will be performed by recording a representative parameter of volume in the aerobic and resistance training components. In aerobic training, the volume will be represented by the distance covered on the treadmill. In the resistance component, the volume will be represented by the number of repetitions (average between sets and/or total) in the sitting and standing exercise in phase 1 and in the leg press in phase 2. These load quantifications will be performed in all sessions of the intervention.

The SpO_2_ will be assessed by finger oximetry, during and after all exercise sessions, by pulse oximeter (HC261 Multilaser) throughout phase 1 and at least before sessions in phase 2 of the rehabilitation programme.

For safety, blood pressure will also be measured before all mesocycle 1 exercise sessions and will continue to be measured before all sessions in patients who have hypertension or some hemodynamic alteration. The evaluation will be performed using automatic equipment (OMRON, model HEM-7113, Brazil), following the procedures described by Barroso [23]. For safety, patients who have blood pressure values [3] 160 mmHg for systolic blood pressure and/or [3] 105 mmHg for diastolic blood pressure will not be able to start the exercise session. Additionally, to analyse the acute effect of exercise, blood pressure values will be measured immediately, 15 and 30 min after the first and last session of each training mesocycle.

Capillary blood glucose collections will be performed, primarily for safety, only in patients with glycaemic alterations. The number of assessment sessions will be defined individually. For exercise purposes, glycaemic values will be measured before, immediately, 15 and 30 min after the first and last session of each training mesocycle. These collections will be performed by means of a digital puncture with disposable lancets (*Accu-Check Safe-T-Pro Uno,* Roche, Portugal) and a drop of capillary blood will be used to fill a test strip that will be analysed in a clinical glucometer (*Accu-Check Performa,* Roche, Portugal) that assesses the blood glucose concentration at the time of collection in approximately 5s.

The affective response to the exercise will be determined from the sensation scale of Hardy and Rejeski [24]. This instrument is composed of an 11-point scale, ranging from +5 (“very good”) to −5 (“very bad”). To use this instrument, at the end of the first and last session of each mesocycle, participants will answer the following question: “How pleasant was it for you to perform this exercise session?”

### Randomisation

Patients included in the study will be randomly allocated to one of the two groups. One group will undergo a clinical and functional rehabilitation programme, and the other group will be considered a control, without exercise intervention.

The allocation list will be concealed from all outcome assessors. In order to balance the groups in terms of number of participants, as well as between men and women, a 1:1 block randomisation will be used, stratified by sex. The randomisation process will be carried out using an online software *www.randomizer.org*. This stage will be performed by a researcher who will not be involved in the other experimental procedures of the study.

After baseline evaluations of each patient block, one manager of the study will send the code identifiers of the patients for randomisation by e-mail. This same manager receives by e-mail the allocation list and informs the exercise training providers, while the other manager, responsible for the contact with patients, informs the patients individually about allocation and corresponding procedures (starting training or control procedure meeting).

### Functional capacity

The assessments of functional capacity will be performed at baseline, at week 12 and after 24 weeks of intervention. The functional indicator of cardiorespiratory fitness and the primary outcome of the study will be evaluated by the 6-min walk test, following the protocol of the American Thoracic Society [13], which consists of measuring the total walking distance in a predetermined area of 30 m long during 6 min. During the test, the patient will be encouraged by the evaluator through standardised incentive phrases, every minute respectively: “There are 5 min left to finish the test. Keep the pace”; “There are 4 min left to finish the test. Keep the pace”; “You’ve already taken half the test. Keep the pace”; “One min. to finish the test. Keep the pace”; and finally, when the timer reaches zero or reaches 6 min, “Stop”. Every 2 min, during the patient’s walking, the values of the distance covered, HR, SpO_2_, dyspnea effort and RPE will be recorded. The test will be stopped immediately if the patient reports chest pain, intolerable dyspnea, leg cramps, staggering gait, diaphoresis and a pale or grey appearance, or if there is a decrease in SpO_2_ of more than 4%. If the test is stopped for any of these reasons, the patient should be seated or lie supine. After the test is properly completed, the number of laps completed will be recorded as well as the total distance covered, blood pressure (systolic and diastolic), O_2_ saturation, heart rate, dyspnea effort (Borg Scale CR-10) and lower limbs (LL) effort (Borg Scale CR-10). The referred physiological and effort measures will be evaluated also in the first, second, third and sixth minute of recovery. After 15 to 20 min of recovery, the test is performed again, following the same protocol, adopting the better score as the final outcome.

Functional mobility (dynamic balance and agility) will be evaluated by the Timed-Up-and-Go (TUG) test. The TUG will follow the following protocol: from a sitting position in a chair (with back supported), the subjects will get up from it (without the help of hands), walk and go around a Chinese demarcation plate positioned on the ground 3 m away and will sit again (with their backs supported). Two attempts will be made at each of the speeds: maximum (TUG-m) and usual (TUG-h), with a 1-min interval between each attempt, with the shortest time for each speed being recorded [25].

The muscular aerobic capacity of the lower limbs will be evaluated by a sit and stand test of 30 s, following the battery protocol of Rikli and Jones (2013) [26], measuring the number of repetitions performed. Flexibility will be evaluated by the sit and reach test, using the Wells bench, by following protocol: participants should sit facing the referred instrument with their knees fully extended and jointed, placing one hand on the other, leaning the trunk forward and reaching as far as possible with the fingertips on the ruler, not being allowed to flex the knees. Each participant will have two attempts, and the highest value achieved will be recorded. Four different scales involving functional status will be applied, which are as follows:

- Post-Covid-19 Functional Status (PCFS), Portuguese version, is an ordinal scale, containing six response levels, comprising the full range of functional outcomes, focusing on limitations of tasks and activities of daily living, both at home, work/study, as well as lifestyle changes [27].
- Modified Medical Research Council (MMRC) consists only of a question about shortness of breath at different levels of activity, being scored from 0 (best clinical condition) to 4 (worst clinical condition) [28].
- Tilburg Frailty Indicator is a multidimensional assessment questionnaire for frailty in the elderly, consisting of 15 questions, distributed in physical, psychological and social domains [29].
- Sarcopenia Form (SARC-F) involves five questions about strength components, need for assistance when walking, getting up and sitting in a chair, climbing stairs and history of falls. The scoring scale has three levels from 0 to 2 points for each item, with the total scoring range being between 0 and 10 [30].
- Chalder Fatigue Scale consists of seven questions addressing physical fatigue and four questions addressing mental fatigue. The answers should be understood by the frequency of occurrence (never, rarely, sometimes and always) [18].
- Post-Exertion Malaise Scale addresses five questions about the individual’s sensations in relation to physical exertion. Questions should be answered regarding the frequency (never, a little, half the time, most of the time, always) and the severity (absent, mild, moderate, severe, very severe) in which they occur [31].
- The Patient-Specific Functional Scale (PSFS) was developed especially for patients with musculoskeletal disorders. It consists of an interview in which the patient chooses five important activities whose execution is difficult or impossible due to his disability or health problem. For each activity, the patient assigns a difficulty score from 0 (“unable to perform the activity”) to 10 (“able to perform the activity at the same level as before the injury or problem”). In subsequent reassessments, the patient re-scores the initial activities or identifies new activity limitations that have arisen over time [32].

### Neuromuscular outcomes

Isometric handgrip strength will be measured using the Carci^@^ handgrip dynamometer (CEFISE, São Paulo, SP) at baseline and after 24 weeks of intervention. Three measurements will be performed on each hand, with a 1-min interval among them, the highest value being recorded.

To assess the isometric force production capacity from the elbow and knee flexor and extensor muscle groups, a micro FET2 HHD manual dynamometer (Hoggan Health Industries, Salt Lake City, Utah) will be used. For the measurement, it will be necessary for the participant to exert force against the dynamometer that indicates the result of the force produced in newtons (N). The maximum isometric contraction values will be recorded. Measurements will be made on the three muscle groups bilaterally, and the highest value of three contractions will be recorded. An interval rest of 1 min will be applied between each contraction in order to avoid muscle fatigue.

To determine the 90° angle of the position of the elbow, knee and hip joints, a universal goniometer with two 20-cm-long rods and a fulcrum with a measurement precision of 2° (*Trident Gon-pvc*) will be used. The sitting position will be adopted for knee assessment, and the lying position will be adopted for the elbow and hip assessment. The individual being evaluated will sit on a stretcher, on which their feet will not touch the floor.

### Anthropometry and body composition assessments

Anthropometric and body composition measurements will be performed at baseline, at the 12th week and after 24 weeks of intervention. Such measurements will always be carried out in the morning on an empty stomach (at least 4 h of fasting). All participants will be instructed not to perform physical exercise the day before data collection and to abstain from alcoholic, caffeinated and other diuretic beverages in the 48 h preceding the test. Participants will be instructed to wear gym clothes, without zippers or metal, to be barefoot, without earrings or rings or any type of metal, and urinate 30 min before the assessment [33]. In addition, the participants will be asked to inform researchers about the use of a cardiac pacemaker or bone prosthesis, and the use of diuretic or calcium-related drug. After completing the assessments, participants will receive a snack, which will consist of a package of highly nutritious cookies (25 g) and a banana.

The body mass (multifrequency Bioimpedance InBody® 720 model, Biospace, Los Angeles, USA) and height (Stadiometer, Alturaexata®, with 1 mm precision) will be recorded and their data will be used to determine the body mass index (BMI). Waist circumference will be measured (flexible and inelastic measuring tape, Cescorf®, with a precision of 1 mm) and with it, the waist-to-height ratio will also be determined.

Computed densitometry by dual energy radiological absorptiometry (DXA), Hologic®, Discovery WI Fan-beam model - S/N 81593 (Hologic Inc., Bedford, Massachusetts, EUA) will be used to assess fat mass, fat-free mass, lean mass, bone mineral density and visceral adipose tissue, expressed in absolute and relative terms. The device will be calibrated before the analyses and will be used following the manufacturer’s recommendations. The assessment will be done in automatic and whole-body mode.

The phase angle (PhA) will be estimated using a multifrequency Bioimpedance InBody® 720 model (Biospace, Los Angeles, USA), with eight electrodes, measuring resistance at five frequencies (1, 50, 250, 500 and 1000 kHz) and reactance in three (5, 50 and 250 kHz). To estimate the PhA, the raw values of the BIA will be used: resistance (R) and reactance (Xc). The technique provides impedance (Z) and Xc data at a frequency of 50 kHz, and from these, the R value will be calculated by the proportional sum of the body, in which the upper limbs represent 40% of the total body R, the trunk represents 10% and the lower limbs represent 50%. Thus, the PhA, expressed in degrees (°), will be calculated through the equation: PhA = Arctangent (Xc / R) × (180°/ π) [34]. The device will be calibrated and used according to the manufacturer’ recommendations. In addition, from Bioimpedance, data on total body water (intra- and extracellular) and basal metabolic rate are also obtained.

### Respiratory outcomes

Respiratory outcomes will be assessed at baseline and after 24 weeks of intervention. Pulmonary function assessment will be performed following the procedures recommended by the American Respiratory Society (ATS) and the European Respiratory Society (ERS) for the respiratory oscillometry [35], spirometry (slow manoeuvre) [36] and single-breath carbon monoxide uptake in the lung (https://erj.ersjournals.com/content/49/1/1600016). The equipment will be calibrated daily before carrying out the assessments with a 3-L syringe according to the specifications of the manufacturers [37].

Respiratory system impedance (Zrs), resistance of respiratory system at 5 Hz (Rrs5), reactance of respiratory system at 5 Hz (Xrs5) and area of reactance (AX), in kPa/L/s, will be measured before and after the administration of the bronchodilator salbutamol (400 μg) using the impulse oscillometry system (IOS) MasterScreen JAEGER™ (Germany) [36]. The ratio between inspired volume and vital capacity (VI/VC) in %, diffusing capacity of the lungs for carbon monoxide (DLCO) in mL/min/mmHg, alveolar volume (VA) in L and transfer coefficient of the lung for carbon monoxide (KCO) in mL/min/mmHg/L will be measured using the Vmax® VIASYS Respiratory Care Inc (USA). The values of DLCO, VA and KCO will also be calculated in % predicted [38].

### Cardiovascular outcomes

Clinical blood pressure, heart rate variability and vascular function will be assessed, all at rest, at baseline, at week 12 and after 24 weeks of intervention. Blood pressure measurements at rest will be performed on two non-consecutive days, using automatic blood pressure monitoring equipment (OMRON, model HEM-7113, Brazil), with cuffs appropriate to the circumferences of the participants’ arms, following the recommendations of the Brazilian Guidelines on Hypertension [39]. Therefore, on each of the 2 days, the participants will remain at rest, sitting in a calm environment for 10 min, and then three measurements will be performed, with a 1-min interval between them. For analysis purposes, the average of all measurements performed on each measurement day will be adopted.

Heart rate variability (HRV) will be measured by collecting R-R intervals, beat-to-beat, using a Polar heart rate monitor, Vantage V2 model. Participants must remain at rest in a supine position for 10 min, and later the recording of R-R intervals will be performed for another 10 min. This data will initially be exported to the Polar Flow programme from the Polar Flow Sync software.

Subsequently, the data will be filtered to eliminate possible noise from ectopic beats or device reading errors in the order of 20 bpm [40], and the percentage of correction of the R-R intervals cannot exceed 2%. This procedure will be performed in Polar Precision Performance software, version 4.03. In the decomposition of the parameters provided by the HRV, the respective R-R intervals will be exported to the HRV Analysis Software – Kubios programme (The Biomedical Signal Analysis Group, University of Kuopio, Finland) and the analyses will be performed in the time and frequency domains. In the analysis of the frequency domain, the Fourier transform is used to quantify the low-frequency (LF: 0.04–0.15 Hz) and high-frequency (HF: 0.15–0.4 Hz) bands, in units normalised, following the recommendations of the Task Force of the European Society of Cardiology and the North American Society of Pacing and Electrophysiology [41].

Prior to blood pressure and HRV collections, patients will receive the following instructions: 24 h without practising moderate or vigorous physical activity; 12 h without ingesting alcoholic beverages, teas, coffee, coca cola, or any beverage or food containing caffeine; take medication as usual (if in the morning, take it before coming in; if at night, take it the night before); maintain light and usual food the day before and on the day of collection. In addition, on collection days, it will be recommended that the patient empty their bladder immediately prior to assessment, if necessary.

Vascular function will be assessed using the flow-mediated dilation (FMD) technique using the LOGIQ S7 Expert Ultrasound device (GE Healthcare), following the recommendations of Thijssen [42]. Before the start of collection, individuals will receive the following guidelines: (a) rest in a quiet and dark room for a period of 10 to 15 min, (b) avoid exercise for at least 24 h, (c) avoid alcohol and foods or beverages that contain caffeine or are rich in polyphenols for at least 12 h and (d) maintain a light and habitual diet the day before and on the day of collection.

Individuals will remain lying down in dorsal decubitus, with the arm extended at heart level for the evaluation of FMD. The measurement will be performed in the brachial artery on the right side. At that moment, a cuff will be placed on the subject’s forearm, 5 cm from the antecubital fossa. The ultrasound should be adjusted for vascular analysis of the brachial artery. The transducer will be positioned over the brachial artery, seeking to obtain the best possible image in B-mode. From there, the ultrasound parameters must be configured so that the image is obtained with optimal quality. Then, the Doppler function will be activated to capture the blood flow together with the image of the artery in real time.

For the collection of the FMD, the diameter and the blood flow of the artery baseline will be evaluated before the inflation of the cuff, for a period of at least 30 s. The cuff will then be inflated to 50 mmHg above the individual’s systolic BP for 5 min, after which the cuff will be deflated, and diameter and blood flow measurements will be evaluated for 3 min. Post-deflation diameter measurement will begin 30 s prior to cuff release. Blood velocity will be assessed using an insonation of 60° or less. During the entire collection, continuous measurements of the flow velocity and the diameter of the brachial artery, through the Doppler function, will be recorded for future analyses. Pre- and post-intervention assessments will be standardised and performed at similar times of day.

Data analysis will be performed using continuous edge detection and wall-tracking software, and automated mathematical algorithms will be used to calculate peak diameter. The baseline diameter and the FMD response in absolute (mm) and relative (%) changes will be presented.

### Cardiorespiratory fitness

A maximal incremental cycle ergometer test (Lode Excalibur Sport, Lode BC, Groningen, Netherlands) will be performed at baseline and after 24 weeks of intervention. The initial workload will be 20 W maintained during 4 min, followed by 10 W increments every 30 s until voluntary exhaustion. Participants will be instructed to maintain cadence at 65–75 rpm throughout the test. The test will end when the cadence drops more than 10 rpm for more than 5 s. The VO_2_ will be measured breath by breath throughout the procedure from the expired gas (COSMED, model QUARK PFT ERGO, Rome, Italy), with subsequent reduction of the data to averages of 15 s. The VO_2_ peak will be considered as the highest value obtained during the test in the 15-s intervals. The QUARK PFT ERGO system will be calibrated before each test to ensure accurate measurements of ambient air, cylinder gas, turbine and delay, in accordance with the manufacturer’s recommendations. The peak power (PP) will be considered the highest workload reached during the test.

HR monitoring will be performed by means of an HR monitor incorporated into the gas analyser, allowing the recording and storage of HR behaviour synchronised with VO_2_. To consider the tests as maximum, the criteria proposed by Basset and Howley [43] will be adopted. The ventilatory threshold (LV1) will be determined as follows: (a) the moment in which a first non-linear increase in the carbon dioxide production ratio will occur (VCO_2_) versus VO_2_ (v-slope method); (b) an increase in minute ventilation (VE) relative to VO_2_ (VE/VO_2_) without an evident increase in VE/VCO_2_; and (c) a first increase in the final tension of O_2_ without a drop in the final tension of CO_2_ [44]. The second ventilatory threshold (LV2) will be determined by the increase in minute ventilation (VE) relative to VCO_2_ (VE/VCO_2_) without an evident increase in VE/VO_2_ [45].

### Physical activity levels and sedentary behaviour

Habitual physical activity and time spent in sedentary behaviour will be evaluated at baseline and after 24 weeks of intervention as behavioural parameters, based on accelerometry, using devices of the GT3X and GT3X+ models (Actigraph Pensacola, FL, USA) and Actilife software (Actigraph Pensacola, FL, USA). Each participant will be instructed to use the accelerometer for seven consecutive days, removing it only to sleep, shower or perform water activities. The device will be placed on an elastic belt and fixed on the right side of the hip. Data will be collected at a frequency of 30 Hz and analysed in 60-s epochs. Periods with consecutive zeros for 60 min or more (with a 2 min tolerance) will be interpreted as non-use time and excluded from the analysis. For analysis purposes, a minimum of 10 h of daily activity recordings, for at least 4 days, three weekdays and one weekend day will be considered valid data. The average time spent in each intensity of physical activity and in sedentary behaviour will be calculated from the cut-off points proposed by Freedson et al. [46] and Sasaki et al. [47]. Data will be analysed in minute/day, adjusted for number of days of use and daily time of use. From this, the following outcomes will be analysed: time in sedentary behaviour (minute/day), in light physical activity (minute/day), moderate (minute/day) and vigorous (minute/day), time in total physical activity (minute/day), total number of sedentary behaviour breaks and average duration of breaks (minute/day), total number of bouts of moderate to vigorous physical activity and average duration of bouts of moderate to vigorous physical activity (minute/day).

### Psychosocial outcomes

As psychosocial parameters, at baseline, at the 12th and after 24 weeks of intervention, the following will be evaluated: perception of health and quality of life, level of anxiety and depressive symptoms and quality of sleep.

The perception of health and quality of life will be evaluated by the EQ-5D-5L instrument, which consists of a page with an objective questionnaire and another page that is a visual analogue scale. The first page consists of five dimensions: mobility, self-care, usual activities, pain/discomfort and anxiety/depression. Each dimension has five levels, ranging from “no problem” to “extreme problem”. The scale is used for the participant to record their general perception of health on a vertical scale, ranging from 0 (“worst health”) to 100 (“best health”).

Anxiety and depression levels will be evaluated by the Hospital Anxiety and Depression (HAD) scale, composed of 14 items, with four possible answers [48, 49]. In addition, the Patient Health Questionnaire (PHQ-9) will be used, an instrument that allows screening for depressive symptoms in adults, consisting of a questionnaire composed of nine questions that assess the presence of symptoms of depression described in the Diagnostic and Statistical Manual of Mental Disorders (DSM-IV). The nine symptoms consist of depressed mood, anhedonia (loss of interest or pleasure in doing things), problems with sleep, tiredness or lack of energy, change in appetite or weight, feelings of guilt or worthlessness, problems concentrating, being slow or restless and suicidal thoughts [50].

Sleep quality will be assessed using the Pittsburgh Sleep Quality Index (PSQI-BR) [51], which consists of a questionnaire consisting of 19 self-administered questions, plus five questions to be answered by the bed partner. The PSQI contains seven domains: subjective sleep quality, sleep latency, sleep duration, habitual sleep efficiency, sleep disturbances, sleep medication use and daytime dysfunction [51].

### Barriers to physical activity

Personal and environmental barriers to physical activity will be obtained using a validated questionnaire for adults [31]. From a list of 19 reasons for not exercising (more), the participant should mark all that apply for them. Personal barriers include the following: “laziness and fatigue (tiredness, physical exhaustion)”, “lack of motivation”, “lack of time”, “lack of partner”, “pain, injury”, “I am not able to”, “I have no way to commute for exercising”, “It is not fun”, “need to relax and rest”, “lack of money”, “lack of competence”, “fear of injuries”, “lack of support”, “I do not understand why it is important” and “body shame”. Environmental barriers include “due to the COVID-19 pandemic”, “lack of appropriate facilities/equipment/space” and “weather”.

### Adverse events and intervening factors

From the medical screening, eligibility criteria, monitoring and safety measures described, and the structure of the rehabilitation programme, we believe that the risk for adverse events, especially serious ones, is minimal. Furthermore, physical tests of greater cardiovascular overload will only be performed in a hospital environment or in the presence of a physician, for greater patient safety. However, if any event occurs, the patients will be treated immediately by the team involved in the research.

During the intervention, patients who are in the intervention group will respond every Friday to a recall questionnaire of adverse events of the last 7 days. Patients will answer simple questions about possible events, unusual physical activity, medication changes, diagnoses and medical care, pain related or not to physical activity, and general well-being. Patients in the control group will be contacted via video call every 6 weeks to control adverse events and will answer the same questionnaire applied weekly to the intervention group.

The possibility of discontinuation of the study will be discussed by the three coordinators (R.S.D, A.M.G and R.M) in the event of a serious adverse event or a succession of mild and moderate adverse events.

### Sanitary measures

All assessments and physical training sessions will respect the recommended health measures to prevent the spread of COVID-19. Especially in relation to the intervention, it is important to note that the exercise sessions will be held in a ventilated environment and all participants (patients and staff) will be required to wear masks.

Disposable masks will be made available to participants so that they can change them whenever they are wet, and a distance of 1.5 m will be respected between participants and members of the research team. The training providers involved in the project will use PFF2 masks. In addition, all participants must wash their hands before the start of training and will have free access to hand sanitiser throughout all sections. There will be a break (between 20 and 30 min) between classes for complete cleaning of the training place and the equipment involved in the practices.

### Ethical considerations

This project has been approved by the Ethics Committee in Research with Human Beings of the Federal University of Santa Catarina, under protocol number 49487721.9.0000.0121. All participants will be duly informed about all study procedures and, after agreeing to participate, participants will sign the free and informed consent form. In the consent form, the risks and benefits of the study are well delineated. In summary, patients will be informed that participation in the study involves a minimal probability of risks and discomforts, especially by the professional prescription and conduction of the exercise sessions. The research team, consisting of physicians, exercise professionals and other health professionals, will be available for solving any adverse events. Sanitary measures are also made clear. The common discomforts during and after the exercise sessions, such as fatigue, are also explained to patients, which may require reducing the intensity or stopping the exercise.

Any important modification to the protocol that is needed will be discussed among coordinators and managers in monthly meetings and reported to the evaluation team, the ethics committee and the trial registry (ReBEC).

### Coordination and management of the trial

Rodrigo Sudatti Delevatti, Aline Mendes Gerage and Rosemeri Maurici comprise the trial’s coordinating team. While Rosemeri Maurici coordinates the clinical triage of patients, Rodrigo Sudatti Delevatti and Aline Mendes Gerage coordinate the outcome assessment team, the team responsible for patient allocation and the intervention team. The coordinating centre is located in the rehabilitation centre of the sports centre (CDS) of UFSC. There is no specific steering committee or endpoint adjudication committee for this trial. The roles and responsibilities from these committees also belong to the coordination centre. The trial has three managers, Angelica Danielevicz, Maria Eduarda de Moraes Sirydakis and Paulo Urubatan Melo, who will carry out part of the recruitment, perform the scheduling and logistical organisation of the evaluations.

### Data management

The outcome assessors will perform a first treatment of the data when necessary, save a file and send the data to the trial managers. Meetings between the study management and the evaluators will be held to define the form and dates for sending the data. With the data in hand, all will be tabulated in a standardised way in a central folder of the trial, which will be evaluated and organised by the coordinators and managers.

### Data monitoring

There is no specific monitoring committee for this trial. All data are monitored by specific evaluation teams for each outcome group (i.e. cardiovascular outcomes team, functional outcomes team) and sent to trial managers, who check and organise the central database. It is important that this trial is unfunded, and all researchers have no conflicts of interest.

### Analysis plan

Continuous sample characterisation variables will have their normality and homogeneity tested by the Shapiro-Wilk and Levene tests, respectively. Those with normal distribution will be described by mean and standard deviation, and the others will be described by median and interquartile range. The categorical variables of sample characterisation will be described by absolute frequency (*n* sample) and relative (%).

To analyse the effects of the rehabilitation programme, generalised estimation equations will be used, adopting the Bonferroni post hoc. Outcomes will be analysed in two ways (by protocol [PP] and intention to treat [ITT]). For this, patients who complete at least 70% of the proposed sessions will be included in the PP analysis. For ITT analysis, all available data of all patients will be used. For this, all patients will be invited for endpoint evaluations, independently of adherence. For missing data, multiple imputation considering the maximum likelihood estimation approach will be used. Effect sizes (ES) will be calculated using Cohen’s *d* test (Cohen 1988), being considered as small values (0.20 ≤ *d* < 0.50), medium (0.50 ≤ *d* < 0.80) and great (*d* ≥ 0.80).

The significance index adopted will be 0.05. The statistical treatment of the data will be carried out using SPSS (Statistical Package for the Social Sciences), version 22.0.

### Dissemination policy

The findings of this trial should be disseminated in the following forms:

- Individual reports to participants;
- Manuscript published in peer-reviewed periodicals;
- Summaries with language appropriate for the general population on social media;
- Presentations at scientific conferences;
- Work meetings with health professionals.

**Fig. 3:**
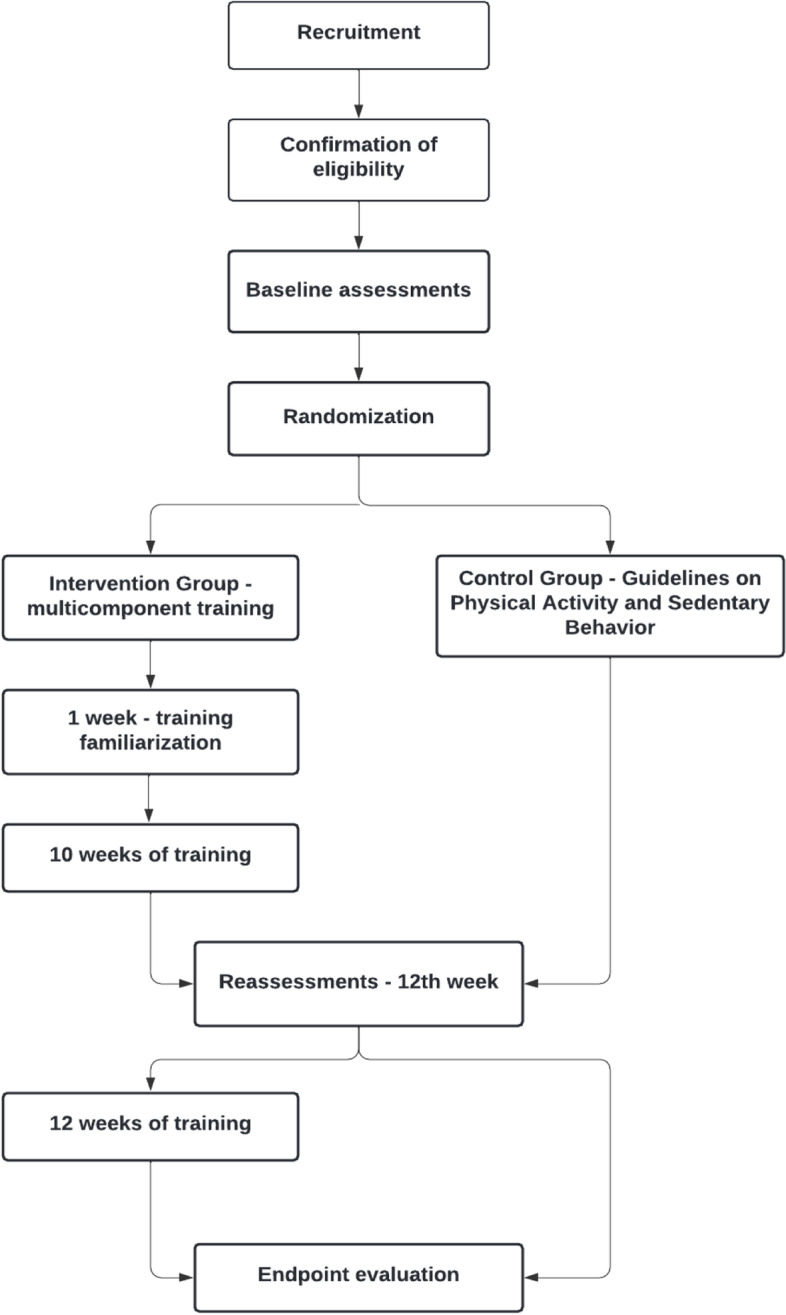
Study flowchart—in article, title of figure below

The flowchart of the study is presented below (Fig. 3).

## Discussion

Despite the millions of deaths that have occurred due to the COVID pandemic in the world, fortunately, the number of patients who have recovered from COVID-19 is increasing. However, a large portion of these patients present with a collection of complications after the acute phase of the disease, which are not necessarily related only to patients who had a severe condition and were hospitalised [52]. There are currently reports that address epidemiological aspects of the so-called syndrome post-COVID-19 or long COVID. As an example of this new context, a cohort study composed of 222 patients who were hospitalised with SARS-CoV-2 infection sought to describe the prevalence of symptoms related to post-COVID-19 syndrome and concluded that 56.3% of patients reported new persistent symptoms or symptoms more than 21 days after diagnosis, and 25% of these patients were unable to return to baseline health status after recovery from the acute infection [53]. On the other hand, an Italian study evaluated 143 patients after hospital discharge after recovery from the acute phase of COVID-19, and they found that 87.4% of these patients reported persistence of at least one symptom 60 days after the onset of the first symptom [54].

Complications related to post-COVID-19 syndrome vary from organ dysfunctions, such as of the heart, lungs and brain [55], to fatigue, headaches, attention disorder, hair loss and dyspnea, which are five among the 55 manifestations considered most prevalent, according to a recent systematic review with meta-analysis [55]. Another important factor related to post-COVID-19 syndrome is the likely psychosocial impact. Reports of persistent malaise and exhaustion similar to chronic fatigue syndrome, in addition to predisposing patients to greater physical weakness, favour the emergence or worsening of emotional disorders and may also leave them susceptible to depression, anxiety, stress disorder, post-traumatic stress disorder and substance use disorder. Additionally, among the complications presented and related to post-COVID-19 syndrome, fatigue is one of the most prevalent, and it greatly contributes to an increase in sedentary behaviour, as the individual is less willing to carry out work activities [56]. This issue should be highlighted as sedentary behaviour contributes to a higher incidence and mortality from cardiovascular disease, cancer, type 2 diabetes, and all-cause mortality [56].

Based on the aforementioned context, it is highly relevant to conduct rehabilitation programmes that provide and enhance the recovery of these individuals, taking into account the complexity existing in the context of COVID-19. We emphasise that our study takes this complexity into account, both in the structuring of the intervention, of a multicomponent nature, as well as in the choice of outcomes, which cover manifestations of the most diverse organic systems, namely behavioural, clinical, morphological, psychosocial and, above all, functional.

As for the prescription of physical training, there is consistent evidence with a rehabilitative approach [56–61] that highlights its supporting therapeutic role in the treatment of numerous diseases and communicable and non-communicable chronic diseases [60], where there is an inverse relationship between regular practice of physical activity and physical exercise and premature mortality [62]. But when talking about rehabilitation programmes in individuals affected by symptoms arising from COVID-19, there is not, to date, sufficient evidence that can guarantee safe recommendations regarding the prescription of physical training, despite the constant emergence of new intervention studies within this perspective. We also emphasise that the literature that addresses both the prescription of exercise training within a biopsychosocial perspective and the aspects inherent to the variables of monitoring and patient safety is limited. Since COVID-19 is a multisystem disease, there is a need for studies that consider the complexity of post-COVID-19 rehabilitation and which are methodologically robust, as in the case of randomised and controlled clinical trials. Of the studies that we have observed so far, all of them present interesting rehabilitation proposals, but at the same time they have important limitations. These limitations range from intervention characteristics (1—short or medium-term intervention periods, 2—only one component/trained physical quality) [19, 63–66] to methodological limitations (1—patient allocation non-randomised, 2—lack of a control group, 3—absence of blinding of the evaluators, 4—absence of details regarding the monitoring of parameters that contribute to patient safety) [ 63, 64, 67].

Concerning the investigation of the effects of physical training on individuals with sequelae from and related to COVID-19, it is relevant to analyse the effect of this training within a multicomponent perspective, especially due to the heterogeneity of these sequelae, which compromise different systems of the human body to different magnitudes. There are a multitude of methods and possibilities for prescribing multicomponent physical training, but in the context of the rehabilitation of post-COVID-19 patients, this training must be especially structured based on the aerobic and resistance components since the physical qualities related to these components are greatly impaired in patients who have experienced COVID-19 in a moderate or severe form. Therefore, conducting an RCT with a multicomponent training intervention, with gradual progression of dosage (volume and intensity) can provide important information about the effectiveness of the present rehabilitation programme.

The design of the present study stands out for the fact that it seeks to test whether the combination of physical training performed from aerobic, balance and resistance exercises, with gradual and individual progression of training loads. Thus, it is expected that the proposed intervention will provide positive results in terms of neuromuscular, respiratory, cardiorespiratory, cardiovascular, behavioural and psychosocial parameters in functional capacity and autonomy and in the quality of life of those people who have not fully recovered from COVI-19 after the phase acute illness. It should also be noted that of the RCTs that aim to evaluate the effect of physical exercise for this population, there is no knowledge to date of the existence of any study that has been conducted for periods longer than 12 weeks. Therefore, it is understood that our study is pioneering in this aspect as it proposes the follow-up of individuals with persistent symptoms of COVID-19 for a period of 24 weeks, who, later, should be tracked in an observational follow-up for at least 5 years.

Based on the aforementioned context, we highlight how relevant it is to measure the long-term impact that physical training can provide to patients in each of the variables that will be analysed. As for the outcomes of our study, we expect that the physical training prescription performed, based on all the previously highlighted criteria, can present promising results even after 24 weeks of physical training supervision, especially regarding our primary outcome (functional capacity according to the 6-min walk test).

We understand that the results of the present study may allow for benefits to be extrapolated which are directly related to the target population of the study; this may have important implications for the optimisation of physical training prescriptions in outpatient rehabilitation programmes.

This clinical trial protocol and its future findings should have important implications. Exercise professionals will be able to use the practical approach adopted for better conduct of its training prescriptions for this population, especially by customisability of protocol. From a theoretical perspective, there is pioneering analysis of the medium (11 weeks) and long-term (24 weeks) effects of the progressive multicomponent training with respect to several important outcomes, using robust methodological control. Clinically, the study will allow a new and safe tool for clinical decision-making, providing data about safety, adherence and the efficacy of multicomponent training in post-COVID-19 scenarios. Finally, the present training protocol, being evidenced as safe and effective, will support the creation of exercise programmes for post-COVID rehabilitation in public health, as well as the performance of other studies with a more pragmatic approach.

### Trial status

Protocol version 1.0, 1 December 2022. The inclusion of patients in the trial will occur in blocks of 6 to 8 patients. The first block was recruited in November 2021 and the last block was recruited in October 2022. All patients included are evaluated for 2 weeks, followed for 24 weeks (intervention period), reavaluated for 2 weeks and included in an observational step, probably with 5 years of duration.

## Supplementary Information

**Additional file 1.** Appendices 1 and 2.

## Data Availability

All materials described in the manuscript, including all relevant raw data, will be freely available to any scientist who wishes to use them for noncommercial purposes, without violating participant confidentiality.
