# Supplementary material for: Effects of physical training on functional, clinical, morphological, behavioural and psychosocial outcomes in post-COVID-19 infection: COVID-19 and REhabilitation study (CORE-study)—a study protocol for a randomised controlled clinical trial
Source: Trials. 2023 Jan 19;24:39. doi: 10.1186/s13063-022-07055-5 (PMC9850322; doi:10.1186/s13063-022-07055-5)
Supplement: Supplementary file 1 — Additional file 1. Appendices 1 and 2. [file 13063_2022_7055_MOESM1_ESM.docx]

**Additional file 1**

**Appendix 1**

FREE AND CLARIFIED CONSENT TERM

**Research title: EFFECTS OF PHYSICAL TRAINING POST-COVID-19 INFECTION ON FUNCTIONAL, CLINICAL AND PSYCHOSOCIAL OUTCOMES: COVID-19 and REhabilitation Study (CORE-Study)**

Responsible researchers: Prof. Dr. Rodrigo Sudatti Delevatti (CDS/UFSC) and Prof. Dr. Aline Mendes Gerage (CDS/UFSC)

Dear Sir/Madam, you are being invited to participate in a research project to be developed by the Federal University of Santa Catarina, whose objective is to analyze the effects of physical training on functional, clinical and psychosocial status in adults after infection by Covid-19. Additionally, we intend to evaluate the association of clinical, functional and psychosocial outcomes with the practice of physical exercises in patients already rehabilitated. This project is based on Resolution 466/2012 of the National Health Council and the researchers are committed to complying with all its items.

**Justification:** COVID-19 provides a varied and complex clinical scenario, with deleterious consequences in various organ systems that affect, among other aspects, the functional capacity of patients affected especially by moderate and severe forms of the disease. In the post-covid-19 infection process, physical rehabilitation is essential and aims to recover the systems affected by the disease and restore patients’ autonomy and quality of life. It is not yet well-established which intervention model is most suitable for this purpose, but it is believed that rehabilitation programmes that include aerobic and strength exercises, in addition to breathing, stretching and balance exercises, may favour important health outcomes, which will be investigated in the present study.

**Procedures:** Upon agreeing to participate in the study, you will be submitted to the following assessments: a) scales (questionnaires) and physical tests that assess your functional capacity, simulating activities of daily living, including, for example, walking, sitting and rising from a chair, the ability to forcefully hold an object with the hands and other parts of the body; b) measurements of weight, height and other parameters of body measurements; c) tests and exams that assess your breathing capacity; d) measurements of blood pressure, heart rate and assessment of the health of your arteries; e) exercise test, on an ergometer, to assess your physical conditioning and the behaviour of your heart and respiratory parameters during the effort; f) blood tests that assess its metabolic and inflammatory profile; g) assessment of the level of physical activity by a portable device to be placed on your waist; h) questionnaires that assess your lifestyle, your quality of life, your sleep quality and some feelings and cognitive and memory capacity. In addition, if you are allocated to the intervention group, you will participate in a physical training programme, which will involve performing physical exercises two or three times a week. If you are allocated to the control group, in addition to receiving a complete report of all assessments to which you are submitted, you will receive instructions on the importance of physical activity and, at the end of the study, you will be invited to participate in a physical rehabilitation programme.

**Risks and discomforts:** The exercise sessions will be conducted by trained professionals, who will properly instruct you on the performance of each activity and will give you all the necessary support throughout the study. In addition, all safety procedures and health measures related to controlling and combating the pandemic will be adopted, in accordance with the guidelines of national and international health organisations. The exercise sessions and all the measures to be carried out in the study are well tolerated and have low risks, but especially at the beginning of the rehabilitation programme you may feel a little tired when performing the exercises. If this occurs, you can ask to decrease the intensity of the exercise or ask to rest and/or stop the effort at any time. In the evaluations, you may experience some discomfort, namely: a slight discomfort in the arm during blood pressure measurements and during blood collection, discomfort in the stress test or in the evaluations of respiratory parameters, the latter two evaluated in a hospital environment. Specifically for two assessments that should take place in a fasting state, the researchers will provide a snack shortly after the end of the collection in order to minimise discomfort associated with fasting and/or hypoglycemia. This snack will be the responsibility and cost of the researchers. If by chance you present any abnormal symptoms/discomfort during any evaluation or during the exercise session, the team involved in the study will provide all the necessary support. In addition, it may happen that you become tired or uncomfortable when answering the survey questionnaires, but in these cases, you can request a break to rest at any time you deem necessary.

**Benefits:** As benefits, you will receive a comprehensive assessment of your functional, clinical and psychosocial health, in addition to the prescription and individualised supervision of physical exercises focused on the rehabilitation of damages caused by COVID-19.

**Confidentiality:** The identity of the participants will be completely preserved, but a breach of confidentiality, even if involuntary and unintentional, may occur. The general results of the research (not related to the participants, without nominal identifications) will be published only in scientific events and publications. The participant will be guaranteed the confidentiality of the data and the right to withdraw from the study when it suits him/her, without any kind of prejudice, and any and all information/questions will be clarified at any time during the study.

**Guarantee of reimbursement and indemnity:** You will not have any type of expense to participate in this research, nor will you receive any financial compensation for this, but in case of expenses demonstrably arising from the research, the right to reimbursement. In addition, in the event of any material or immaterial damage resulting from the research, you will be entitled to compensation as recommended by the current resolution.

After these clarifications, we ask for your consent to participate in this research. Two copies of this document must be signed by you and the researcher in charge, and one of these duly signed copies will remain with you.

Participant’s signature:_________________________________________

Date:___/___/_____

We thank you in advance for your attention and make ourselves available for any clarifications.

Yours sincerely.

______________________________

Prof. Dr. Rodrigo Sudatti Delevatti (UFSC)

Tel: (48) 99108 4365

e-mail: rodrigo.delevatti@ufsc.br

Endereço: Estrada Manoel Leôncio de Souza Brito, nº 650, apto 201N, Vargem Pequena, Florianópolis - SC

Comitê de Ética em Pesquisa com Seres Humanos

Universidade Federal de Santa Catarina - Prédio Reitoria II

R: Desembargador Vitor Lima, nº 222, sala 401, Trindade, Florianópolis/SC. CEP 88.040-400

Contato: (48) 3721-6094

E-mail: [cep.propesq@contato.ufsc.br](mailto:cep.propesq@contato.ufsc.br)

Appendix 2

**SPIRIT Checklist for *Trials***

Complete this checklist by entering the page and line numbers where each of the items listed below can be found in your manuscript.

Your manuscript may not currently address all the items on the checklist. Please modify your text to include the missing information. If you are certain that an item does not apply, please state "n/a" and provide a short explanation. **Leaving an item blank or stating “n/a” without an explanation will lead to your manuscript being returned before review.**

Upload your completed checklist as an additional file when you submit to *Trials*. You must reference this additional file in the main text of your protocol submission. The completed SPIRIT figure must be included within the main body of the protocol text and can be downloaded here:<http://www.spirit-statement.org/schedule-of-enrolment-interventions-and-assessments/>

In your methods section, please state that you used the SPIRIT reporting guidelines, and cite them as:

Chan A-W, Tetzlaff JM, Gøtzsche PC, Altman DG, Mann H, Berlin J, Dickersin K, Hróbjartsson A, Schulz KF, Parulekar WR, Krleža-Jerić K, Laupacis A, Moher D. SPIRIT 2013 Explanation and Elaboration: Guidance for protocols of clinical trials. BMJ. 2013;346:e7586

|  |  | **Reporting Item** | **Page and Line Number** | **Reason if not applicable** |
| --- | --- | --- | --- | --- |
| **Administrative information** | | | | |
| Title | [#1](https://www.goodreports.org/reporting-checklists/spirit/info/#1) | Descriptive title identifying the study design, population, interventions, and, if applicable, trial acronym | Page 1, line 1, 2, 3, 4 |  |
| Trial registration | [#2a](https://www.goodreports.org/reporting-checklists/spirit/info/#2a) | Trial identifier and registry name. If not yet registered, name of intended registry | Page 5, line 108 |  |
| Trial registration: data set | [#2b](https://www.goodreports.org/reporting-checklists/spirit/info/#2b) | All items from the World Health Organization Trial Registration Data Set | Page 5, line 108 |  |
| Protocol version | [#3](https://www.goodreports.org/reporting-checklists/spirit/info/#3) | Date and version identifier | Page 5, line 108 |  |
| Funding | [#4](https://www.goodreports.org/reporting-checklists/spirit/info/#4) | Sources and types of financial, material, and other support |  | n/a There is no funding associated with the project. |
| Roles and responsibilities: contributorship | [#5a](https://www.goodreports.org/reporting-checklists/spirit/info/#5a) | Names, affiliations, and roles of protocol contributors | Pages 1- 4, line 6-82 |  |
| Roles and responsibilities: sponsor contact information | [#5b](https://www.goodreports.org/reporting-checklists/spirit/info/#5b) | Name and contact information for the trial sponsor |  | n/a There is no funding associated with the project. |
| Roles and responsibilities: sponsor and funder | [#5c](https://www.goodreports.org/reporting-checklists/spirit/info/#5c) | Role of study sponsor and funders, if any, in study design; collection, management, analysis, and interpretation of data; writing of the report; and the decision to submit the report for publication, including whether they will have ultimate authority over any of these activities |  | n/a There is no funding associated with the project. |
| Roles and responsibilities: committees | [#5d](https://www.goodreports.org/reporting-checklists/spirit/info/#5d) | Composition, roles, and responsibilities of the coordinating centre, steering committee, endpoint adjudication committee, data management team, and other individuals or groups overseeing the trial, if applicable (see Item 21a for data monitoring committee) |  | n/a There is no committees associated with the project. |
| **Introduction** |  |  |  |  |
| Background and rationale | [#6a](https://www.goodreports.org/reporting-checklists/spirit/info/#6a) | Description of research question and justification for undertaking the trial, including summary of relevant studies (published and unpublished) examining benefits and harms for each intervention | Page 5-6, line 112-136 |  |
| Background and rationale: choice of comparators | [#6b](https://www.goodreports.org/reporting-checklists/spirit/info/#6b) | Explanation for choice of comparators | Page 5, 6, 7, line 137-189 |  |
| Objectives | [#7](https://www.goodreports.org/reporting-checklists/spirit/info/#7) | Specific objectives or hypotheses | Page 8, line 191-194 |  |
| Trial design | [#8](https://www.goodreports.org/reporting-checklists/spirit/info/#8) | Description of trial design including type of trial (e.g. parallel group, crossover, factorial, single group), allocation ratio, and framework (e.g. superiority, equivalence, non-inferiority, exploratory) | Page 8, line 176-190 |  |
| **Methods: Participants, interventions, and outcomes** | | | | |
| Study setting | [#9](https://www.goodreports.org/reporting-checklists/spirit/info/#9) | Description of study settings (e.g. community clinic, academic hospital) and list of countries where data will be collected. Reference to where list of study sites can be obtained | Page 9, line 196-210 |  |
| Eligibility criteria | [#10](https://www.goodreports.org/reporting-checklists/spirit/info/#10) | Inclusion and exclusion criteria for participants. If applicable, eligibility criteria for study centres and individuals who will perform the interventions (e.g. surgeons, psychotherapists) | Page 9-10, line 211-240 |  |
| Interventions: description | [#11a](https://www.goodreports.org/reporting-checklists/spirit/info/#11a) | Interventions for each group with sufficient detail to allow replication, including how and when they will be administered | Pages 12-15, line 261-290 |  |
| Interventions: modifications | [#11b](https://www.goodreports.org/reporting-checklists/spirit/info/#11b) | Criteria for discontinuing or modifying allocated interventions for a given trial participant (e.g. drug dose change in response to harms, participant request, or improving / worsening disease) |  | n/a There is no possibility to modify allocation after randomisation. |
| Interventions: adherance | [#11c](https://www.goodreports.org/reporting-checklists/spirit/info/#11c) | Strategies to improve adherence to intervention protocols, and any procedures for monitoring adherence (e.g. drug tablet return; laboratory tests) | Page 15, lines 292-302 |  |
| Interventions: concomitant care | [#11d](https://www.goodreports.org/reporting-checklists/spirit/info/#11d) | Relevant concomitant care and interventions that are permitted or prohibited during the trial | Page 15-16, line 304-310 |  |
| Outcomes | [#12](https://www.goodreports.org/reporting-checklists/spirit/info/#12) | Primary, secondary, and other outcomes, including the specific measurement variable (e.g. systolic blood pressure), analysis metric (e.g. change from baseline, final value, time to event), method of aggregation (e.g. median, proportion), and time point for each outcome. Explanation of the clinical relevance of chosen efficacy and harm outcomes is strongly recommended | Page 16, line 311-327 |  |
| Participant timeline | [#13](https://www.goodreports.org/reporting-checklists/spirit/info/#13) | Time schedule of enrolment, interventions (including any run-ins and washouts), assessments, and visits for participants. A schematic diagram is highly recommended (see Figure) | Page 7, line 14 |  |
| Sample size | [#14](https://www.goodreports.org/reporting-checklists/spirit/info/#14) | Estimated number of participants needed to achieve study objectives and how it was determined, including clinical and statistical assumptions supporting any sample size calculations | Page 11- 12, line 248-259 |  |
| Recruitment | [#15](https://www.goodreports.org/reporting-checklists/spirit/info/#15) | Strategies for achieving adequate participant enrolment to reach target sample size | Page 10, lines 231-234 | n/a There is no strategies for achieving adequate participant enrolment to reach target sample size. |
| **Methods: Assignment of interventions (for controlled trials)** | | | | |
| Allocation: sequence generation | [#16a](https://www.goodreports.org/reporting-checklists/spirit/info/#16a) | Method of generating the allocation sequence (e.g. computer-generated random numbers), and list of any factors for stratification. To reduce predictability of a random sequence, details of any planned restriction (e.g. blocking) should be provided in a separate document that is unavailable to those who enrol participants or assign interventions | Page 18-19, line 377-390 |  |
| Allocation concealment mechanism | [#16b](https://www.goodreports.org/reporting-checklists/spirit/info/#16b) | Mechanism of implementing the allocation sequence (e.g. central telephone; sequentially numbered, opaque, sealed envelopes), describing any steps to conceal the sequence until interventions are assigned | Page 19, line 381-385 |  |
| Allocation: implementation | [#16c](https://www.goodreports.org/reporting-checklists/spirit/info/#16c) | Who will generate the allocation sequence, who will enrol participants, and who will assign participants to interventions | Page 19, line 386-390 |  |
| Blinding (masking) | [#17a](https://www.goodreports.org/reporting-checklists/spirit/info/#17a) | Who will be blinded after assignment to interventions (e.g. trial participants, care providers, outcome assessors, data analysts), and how | Page 9, line 196-201 |  |
| Blinding (masking): emergency unblinding | [#17b](https://www.goodreports.org/reporting-checklists/spirit/info/#17b) | If blinded, circumstances under which unblinding is permissible, and procedure for revealing a participant’s allocated intervention during the trial |  | n/a Unblinding is not allowed in this research. |
| **Methods: Data collection, management, and analysis** | | | | |
| Data collection plan | [#18a](https://www.goodreports.org/reporting-checklists/spirit/info/#18a) | Plans for assessment and collection of outcome, baseline, and other trial data, including any related processes to promote data quality (e.g. duplicate measurements, training of assessors) and a description of study instruments (e.g. questionnaires, laboratory tests) along with their reliability and validity, if known. Reference to where data collection forms can be found, if not in the protocol | Page 19-30, lines 392-668 |  |
| Data collection plan: retention | [#18b](https://www.goodreports.org/reporting-checklists/spirit/info/#18b) | Plans to promote participant retention and complete follow-up, including list of any outcome data to be collected for participants who discontinue or deviate from intervention protocols |  | n/a There was no plans to promote participant retention and complete follow-up. |
| Data management | [#19](https://www.goodreports.org/reporting-checklists/spirit/info/#19) | Plans for data entry, coding, security, and storage, including any related processes to promote data quality (e.g. double data entry; range checks for data values). Reference to where details of data management procedures can be found, if not in the protocol | Page 32-33,  lines 723-728 |  |
| Statistics: outcomes | [#20a](https://www.goodreports.org/reporting-checklists/spirit/info/#20a) | Statistical methods for analysing primary and secondary outcomes. Reference to where other details of the statistical analysis plan can be found, if not in the protocol | Page 33-34 , line 735-751 |  |
| Statistics: additional analyses | [#20b](https://www.goodreports.org/reporting-checklists/spirit/info/#20b) | Methods for any additional analyses (e.g. subgroup and adjusted analyses) | Page 33-34, line 741-749 |  |
| Statistics: analysis population and missing data | [#20c](https://www.goodreports.org/reporting-checklists/spirit/info/#20c) | Definition of analysis population relating to protocol non-adherence (e.g. as randomised analysis), and any statistical methods to handle missing data (e.g. multiple imputation) |  | n/a There was no analysis population relating to protocol non-adherence. |
| **Methods: Monitoring** | | | | |
| Data monitoring: formal committee | [#21a](https://www.goodreports.org/reporting-checklists/spirit/info/#21a) | Composition of data monitoring committee (DMC); summary of its role and reporting structure; statement of whether it is independent from the sponsor and competing interests; and reference to where further details about its charter can be found, if not in the protocol. Alternatively, an explanation of why a DMC is not needed | Page 32, line 712 |  |
| Data monitoring: interim analysis | [#21b](https://www.goodreports.org/reporting-checklists/spirit/info/#21b) | Description of any interim analyses and stopping guidelines, including who will have access to these interim results and make the final decision to terminate the trial |  | n/a There was no interim analyses and stopping guidelines. |
| Harms | [#22](https://www.goodreports.org/reporting-checklists/spirit/info/#22) | Plans for collecting, assessing, reporting, and managing solicited and spontaneously reported adverse events and other unintended effects of trial interventions or trial conduct | Page 30-31, 669-684 |  |
| Auditing | [#23](https://www.goodreports.org/reporting-checklists/spirit/info/#23) | Frequency and procedures for auditing trial conduct, if any, and whether the process will be independent from investigators and the sponsor |  | n/a There was no frequency and procedures for auditing trial conduct. |
| **Ethics and dissemination** | | | | |
| Research ethics approval | [#24](https://www.goodreports.org/reporting-checklists/spirit/info/#24) | Plans for seeking research ethics committee / institutional review board (REC / IRB) approval | Page 31-32, line 697-700 |  |
| Protocol amendments | [#25](https://www.goodreports.org/reporting-checklists/spirit/info/#25) | Plans for communicating important protocol modifications (e.g. changes to eligibility criteria, outcomes, analyses) to relevant parties (e.g. investigators, REC / IRBs, trial participants, trial registries, journals, regulators) |  | n/a There was no plans for communicating important protocol modifications. |
| Consent or assent | [#26a](https://www.goodreports.org/reporting-checklists/spirit/info/#26a) | Who will obtain informed consent or assent from potential trial participants or authorised surrogates, and how (see Item 32) | Page 32, line 700-711 |  |
| Consent or assent: ancillary studies | [#26b](https://www.goodreports.org/reporting-checklists/spirit/info/#26b) | Additional consent provisions for collection and use of participant data and biological specimens in ancillary studies, if applicable |  | n/a There was no additional consent provisions for collection and use of participant data and biological specimens in ancillary studies. |
| Confidentiality | [#27](https://www.goodreports.org/reporting-checklists/spirit/info/#27) | How personal information about potential and enrolled participants will be collected, shared, and maintained in order to protect confidentiality before, during, and after the trial | Page 32, line 700-711 |  |
| Declaration of interests | [#28](https://www.goodreports.org/reporting-checklists/spirit/info/#28) | Financial and other competing interests for principal investigators for the overall trial and each study site |  | n/a There was no conflict of interests. |
| Data access | [#29](https://www.goodreports.org/reporting-checklists/spirit/info/#29) | Statement of who will have access to the final trial dataset, and disclosure of contractual agreements that limit such access for investigators |  | n/a There was no statement of who will have access to the final trial dataset, and disclosure of contractual agreements that limit such access for investigators |
| Ancillary and post-trial care | [#30](https://www.goodreports.org/reporting-checklists/spirit/info/#30) | Provisions, if any, for ancillary and post-trial care, and for compensation to those who suffer harm from trial participation | Page 36, line 36-41 |  |
| Dissemination policy: trial results | [#31a](https://www.goodreports.org/reporting-checklists/spirit/info/#31a) | Plans for investigators and sponsor to communicate trial results to participants, healthcare professionals, the public, and other relevant groups (e.g. via publication, reporting in results databases, or other data sharing arrangements), including any publication restrictions |  | n/a There was no plans for investigators and sponsor to communicate trial results to participants, healthcare professionals, the public, and other relevant groups (e.g. via publication, reporting in results databases, or other data sharing arrangements), including any publication restrictions. |
| Dissemination policy: authorship | [#31b](https://www.goodreports.org/reporting-checklists/spirit/info/#31b) | Authorship eligibility guidelines and any intended use of professional writers |  | n/a There was no Authorship eligibility guidelines and any intended use of professional writers |
| Dissemination policy: reproducible research | [#31c](https://www.goodreports.org/reporting-checklists/spirit/info/#31c) | Plans, if any, for granting public access to the full protocol, participant-level dataset, and statistical code |  | n/a There was no Plans, if any, for granting public access to the full protocol, participant-level dataset, and statistical code. |
| **Appendices** | | | | |
| Informed consent materials | [#32](https://www.goodreports.org/reporting-checklists/spirit/info/#32) | Model consent form and other related documentation given to participants and authorised surrogates | Pages 48-52, lines 1091-1195 |  |
| Biological specimens | [#33](https://www.goodreports.org/reporting-checklists/spirit/info/#33) | Plans for collection, laboratory evaluation, and storage of biological specimens for genetic or molecular analysis in the current trial and for future use in ancillary studies, if applicable |  | n/a No collection of biological materials. |

It is strongly recommended that this checklist be read in conjunction with the SPIRIT 2013 Explanation & Elaboration for important clarification on the items. Amendments to the protocol should be tracked and dated. The SPIRIT checklist is copyrighted by the SPIRIT Group under the Creative Commons “[Attribution-NonCommercial-NoDerivs 3.0 Unported](http://www.creativecommons.org/licenses/by-nc-nd/3.0/)” license. This checklist can be completed online using https://www.goodreports.org/, a tool made by the EQUATOR Network in collaboration with Penelope.ai
